# Navigation-Guided Thoracolumbar Transpedicular Instrumentation Using the 7D Flash™ Navigation System (SeaSpine) in a Patient With L1 Vertebral Fracture and Spinal Deformity Secondary to Prior Instrumentation for Idiopathic Scoliosis: A Case Report

**DOI:** 10.7759/cureus.92663

**Published:** 2025-09-18

**Authors:** Iturbide A Ponce de Leon Sandoval, Eduardo Callejas Ponce, Carlo E Bañuelos Aluzzi, Eugenio C Robles León, Jorge D Pérez Ruíz, Jacobo Kerbel Sutton, Jose-Carlos Sauri-Barraza

**Affiliations:** 1 Orthopedics and Traumatology Center, American British Cowdray (ABC) Medical Center, Mexico City, MEX; 2 Spine Clinic, American British Cowdray (ABC) Medical Center, Mexico City, MEX

**Keywords:** idiopathic scoliosis, image-guided surgery, pedicle screw instrumentation, seaspine navigation, spinal deformity, spinal navigation, surgical revision, thoracolumbar fracture

## Abstract

We present the case of a 63-year-old woman with a history of spinal instrumentation for adolescent idiopathic scoliosis who sustained an L1 vertebral body fracture after a fall. The patient exhibited sagittal imbalance and flatback deformity, with significant anatomical distortion from prior surgeries. A posterior thoracolumbar transpedicular instrumentation from T12 to L3 was performed using the 7D Flash™ Navigation System (SeaSpine, Carlsbad, CA, USA). Image-guided navigation allowed for accurate screw placement despite complex anatomy, without requiring intraoperative CT. This case highlights the value of spinal navigation in revision settings, particularly for enhancing safety, reducing radiation exposure, and optimizing outcomes in patients with prior instrumentation.

## Introduction

Since the mid-1990s, image-guided spine surgery has evolved significantly, laying a strong foundation for the expansion of advanced surgical techniques such as deformity correction, minimally invasive surgery, and oncologic spine approaches [1]. This technological maturation has transformed the surgical management of complex pathologies by enabling greater precision, reducing morbidity, and improving clinical outcomes. Its effective implementation depends on close collaboration between surgeons and technical teams, with an active role for the surgeon in regulating and adapting these tools to the intraoperative environment [1].

Surgical navigation requires surgeons not only to possess advanced technical skills but also to have a deep understanding of computerized guidance systems and instrumentation. Given the spine's complex anatomy and variability, the use of advanced imaging modalities enhances safety and reduces the need for extensive surgical exposure [1]. Furthermore, from both an administrative and patient safety perspective, any technology that helps reduce postoperative complications and optimizes surgical workflow represents a significant advancement [1].

Recent studies have shown that intraoperative navigation enables precise real-time adjustments of pedicle screw diameter and length, thereby improving implant adaptation to the patient's specific anatomy and reinforcing construct stability [2]. This is reflected in higher screw placement accuracy rates of 91.4% in navigated procedures compared to 86.3% with conventional fluoroscopy (p = 0.034), with an even greater impact in minimally invasive procedures [2]. Additionally, a significantly higher screw diameter to pedicle width (SD/PW) ratio was documented in the navigation group (95.8%) compared to the fluoroscopy group (85.1%), with favorable biomechanical implications for construct stability [2].

Navigation technology has also demonstrated logistical and ergonomic advantages: it allows visualization of critical structures without direct line of sight, reduces surgeon fatigue, and significantly decreases intraoperative radiation exposure. For example, a reduction of up to 75% in radiation exposure time for the surgical team has been reported when comparing navigation to non-navigation groups (13.5 seconds vs. 53.2 seconds, p < 0.01) [3]. This is particularly relevant in spinal surgeries, where higher radiation doses are required compared to limb surgeries due to tissue density and the need for clear imaging [3].

While some studies have not shown direct improvements in clinical outcomes, achieving enhanced radiological safety, precision in instrumentation, and adaptation to anatomical deformities or variations is recognized as a substantial advancement in itself [3]. In this context, computer-assisted navigation not only optimizes technical precision but also redefines the standard of safety and efficacy in modern spine surgery.

Adolescent idiopathic scoliosis (AIS) remains the most common cause of spinal deformity in children and adolescents, often requiring surgical correction in severe cases. Historically, first-generation systems such as Harrington rods offered only one-dimensional correction, often resulting in complications like iatrogenic flat back deformity. Modern pedicle screw-based systems enable three-dimensional correction and lead to improved long-term outcomes. However, AIS correction is still associated with complications such as adjacent segment disease (ASD), proximal/distal junctional kyphosis (PJK/DJK), and degenerative disc disease, which may emerge years after surgery [4]. These alterations, coupled with anatomical distortions from previous instrumentation, present significant challenges in revision or extension surgeries.

In this scenario, surgical navigation proves to be a particularly valuable asset. By enabling accurate trajectory planning and real-time adjustments in altered anatomy, it offers a safer and more effective way to approach re-instrumentation in patients with postsurgical deformities. Navigation facilitates the identification of viable pedicles and avoids critical neurovascular structures, which is especially beneficial in anatomically complex or previously operated spines [1-3].

Additionally, the mechanical implications of long-term spinal instrumentation must not be overlooked, especially in revision surgeries or in patients with prior instrumentation for scoliosis. Stress shielding, a phenomenon in which rigid instrumentation offloads physiological forces away from the fusion mass, can lead to localized osteopenia and mechanical incompetence of the bone [3]. Fractures have been reported at the transition zones between previous and new instrumentation, even in the absence of trauma. This highlights the importance of strategic planning in cases requiring construct extension or hardware removal. In this context, image-guided navigation becomes critical, not only for optimizing screw trajectories and avoiding neurovascular injury but also for reinforcing compromised fusion masses and ensuring structural continuity in mechanically weakened segments [1-3,5].

## Case presentation

This is the case of a 63-year-old female patient who began experiencing her current condition approximately one month prior to presentation at our institution. Her symptoms started after a fall down the stairs, which resulted in incapacitating lumbar pain radiating to both lower extremities. Since then, the pain persisted continuously, significantly limiting her activities of daily living and markedly impairing her quality of life.

The patient has a long-standing history of spinal surgery. In adolescence, she underwent spinal instrumentation for scoliosis, including rod placement and sublaminar wiring, which ultimately resulted in a postsurgical type 2 (global) flatback syndrome. Several years later, the instrumentation was removed. Subsequently, she underwent a lumbar decompression from L4 to S1, which was completed without complications. Subsequently, she received various pain management procedures, including bilateral foraminal injections at L4-L5 and L5-S1, bilateral medial branch blocks from L3 to S1, and a sacral hiatus injection, all of which were uneventful. She eventually underwent an anterior lumbar interbody fusion (ALIF) at L5-S1 with interbody cage placement and bilateral transpedicular fixation. Most recently, she received bilateral medial branch blocks from L1 to S1, again without complications.

The patient presented with axial tenderness on palpation and limited flexion and extension due to pain. Muscle strength was assessed using the Daniels scale, with a score of 5/5 from L2 to S1 bilaterally, according to the American Spinal Injury Association (ASIA) classification. No sensory deficits were observed from L1 to S1 bilaterally. Deep tendon reflexes were normal. Bilateral Lasègue and Bragard tests were performed and were negative.

**Figure 1:**
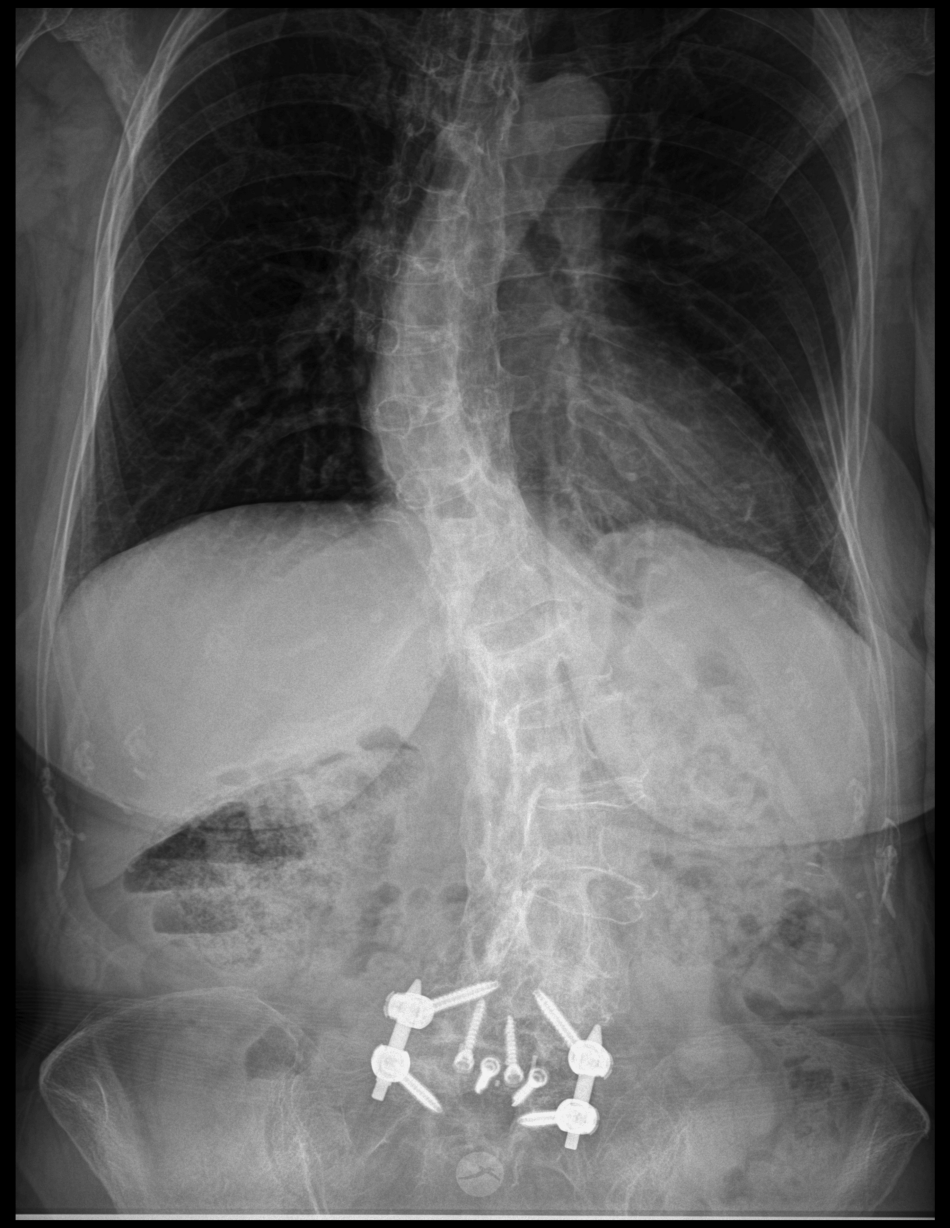
Anteroposterior radiographs of the thoracic and lumbar spine showing a right convex thoracic scoliosis (Cobb angle: 46°) and a left convex lumbar scoliosis (Cobb angle: 40°).

**Figure 2:**
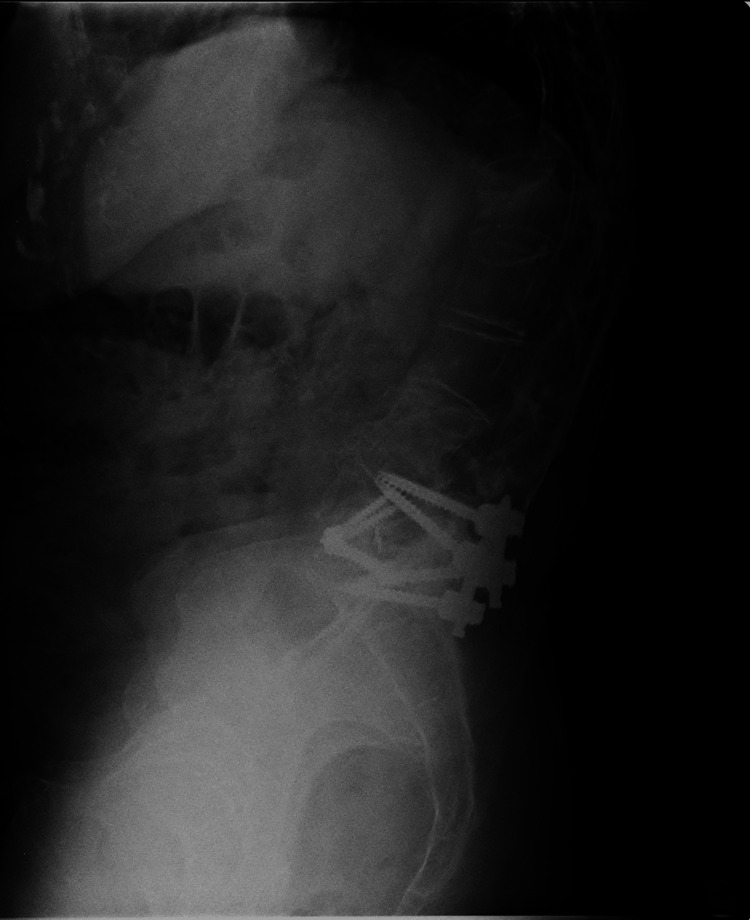
Lateral lumbar spine radiograph showing transpedicular instrumentation at L5–S1 and an interbody cage at the same level, consistent with previous anterior lumbar interbody fusion (ALIF).

**Figure 3:**
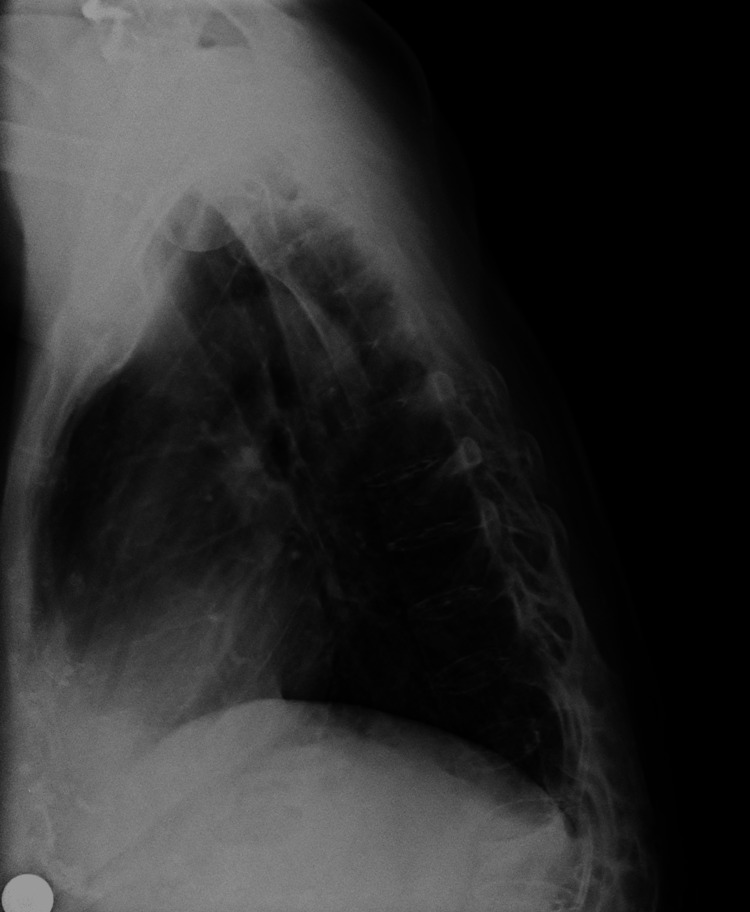
Lateral thoracic spine radiograph demonstrating sagittal imbalance with anterior projection of the trunk, consistent with flat back deformity.

Anteroposterior radiographs of the thoracic and lumbar spine (Figure 1) demonstrated a right convex thoracic scoliosis with a Cobb angle of 46° and a left convex lumbar scoliosis with a Cobb angle of 40°. The lateral lumbar spine radiograph (Figure 2) showed postsurgical changes with transpedicular instrumentation at L5-S1, along with an interbody cage consistent with a prior ALIF at the same level. The lateral thoracic spine view (Figure 3) revealed sagittal imbalance with anterior projection of the trunk, a hallmark feature of postsurgical flat back deformity.

**Figure 4:**
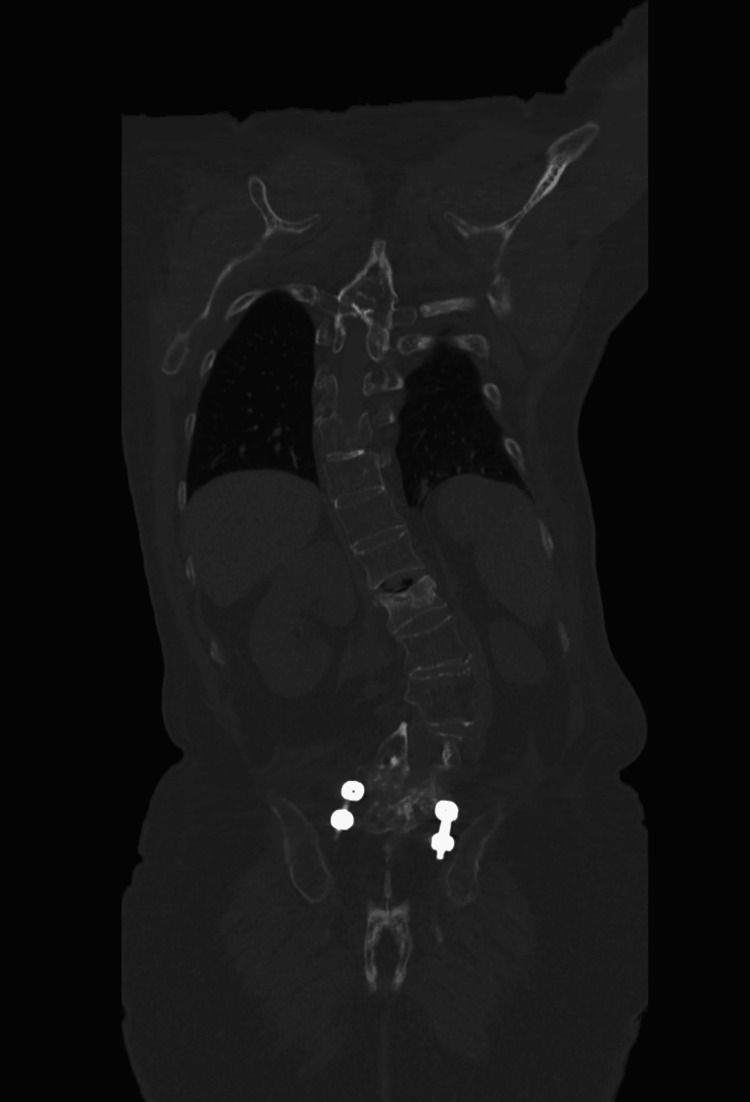
Coronal plane CT image showing collapse of the L1 vertebral body with height loss.

**Figure 5:**
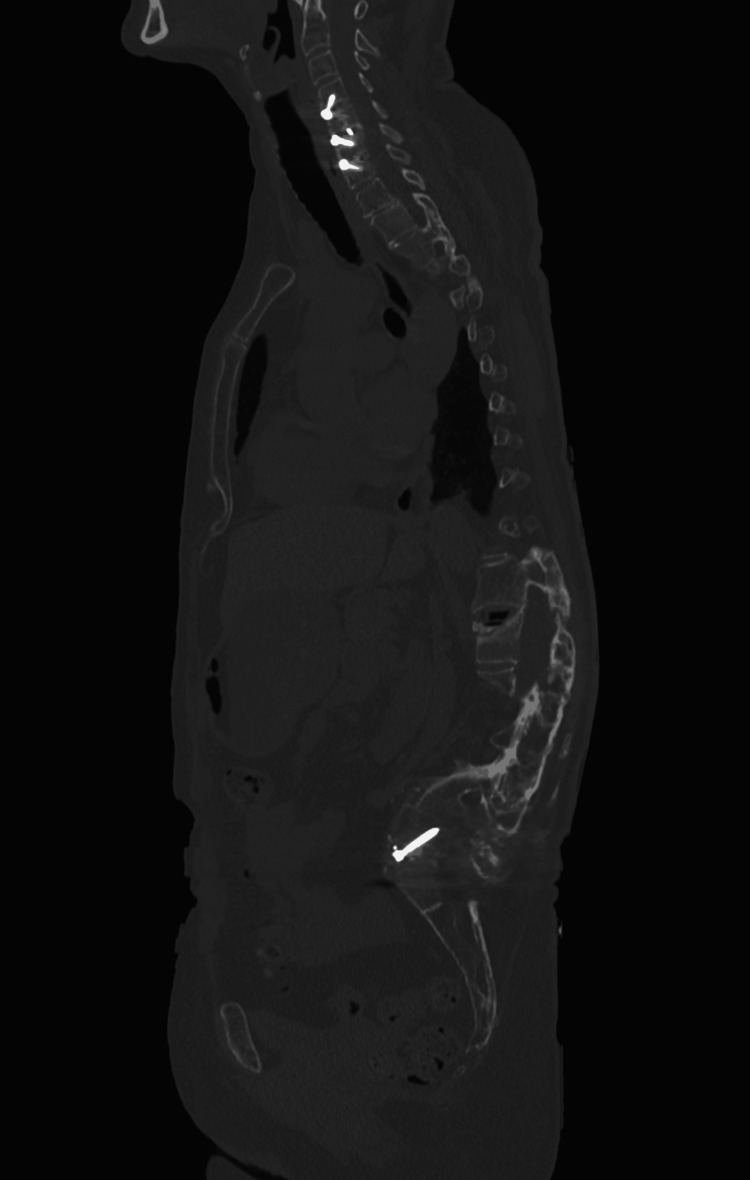
Sagittal plane CT image illustrating L1 vertebral body collapse and postsurgical instrumentation at L5–S1.

**Figure 6:**
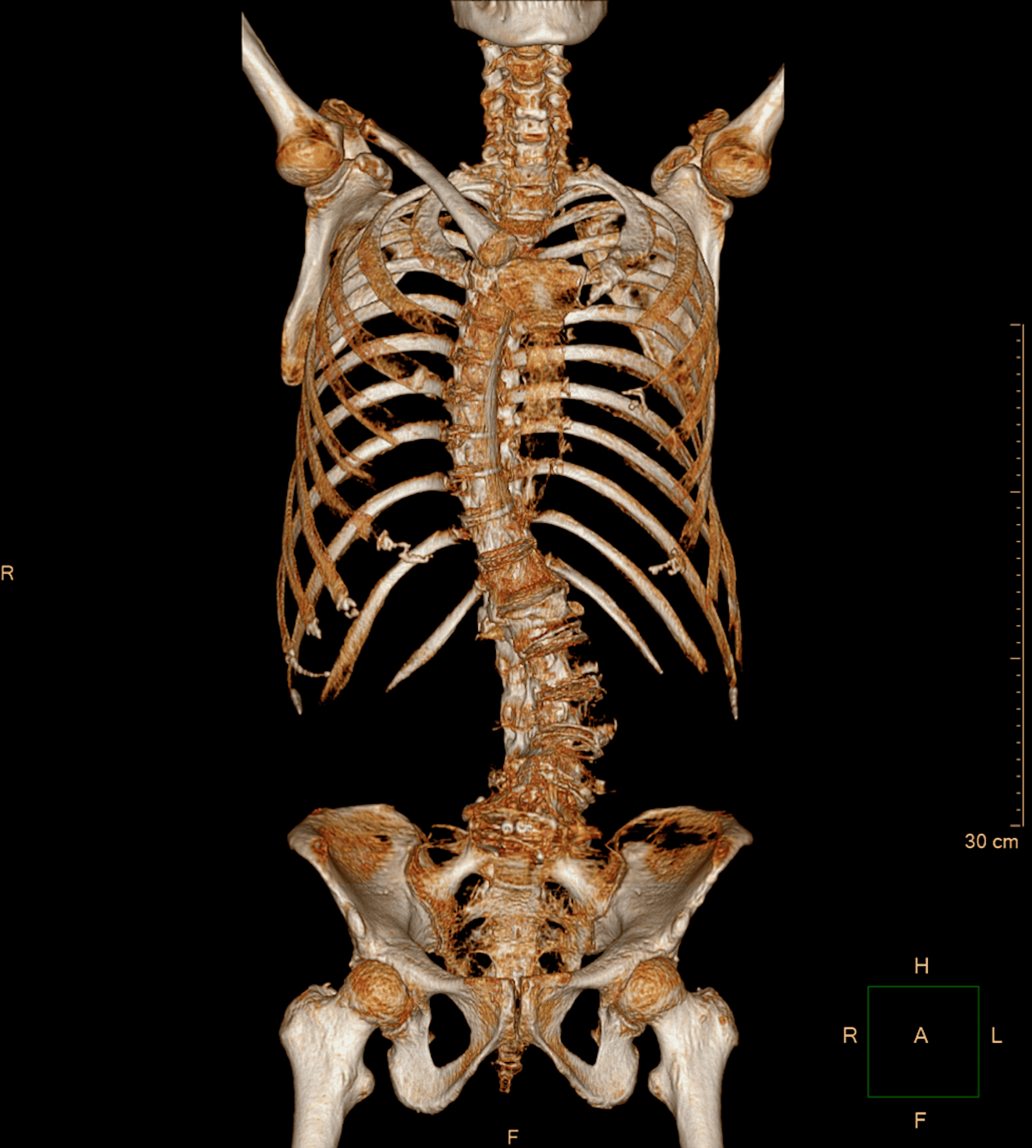
Three-dimensional CT reconstruction demonstrating vertebral collapse at L1 and spinal instrumentation from L5 to S1.

A computed tomography (CT) scan was performed to further evaluate the bony anatomy and surgical instrumentation. Coronal (Figure 4) and sagittal (Figure 5) planes revealed collapse of the L1 vertebral body, with approximately 70% height loss, without evidence of bony fragment intrusion into the spinal canal. Discontinuity was observed in the articular facets of T12, L1, and L2. Postsurgical changes were evident at L5 and S1, including internal fixation hardware consisting of posterior and anterior rods, transpedicular screws, and an intervertebral spacer at the L5-S1 level. A 3D reconstruction (Figure 6) provided a comprehensive visualization of the deformity and instrumentation.

**Figure 7:**
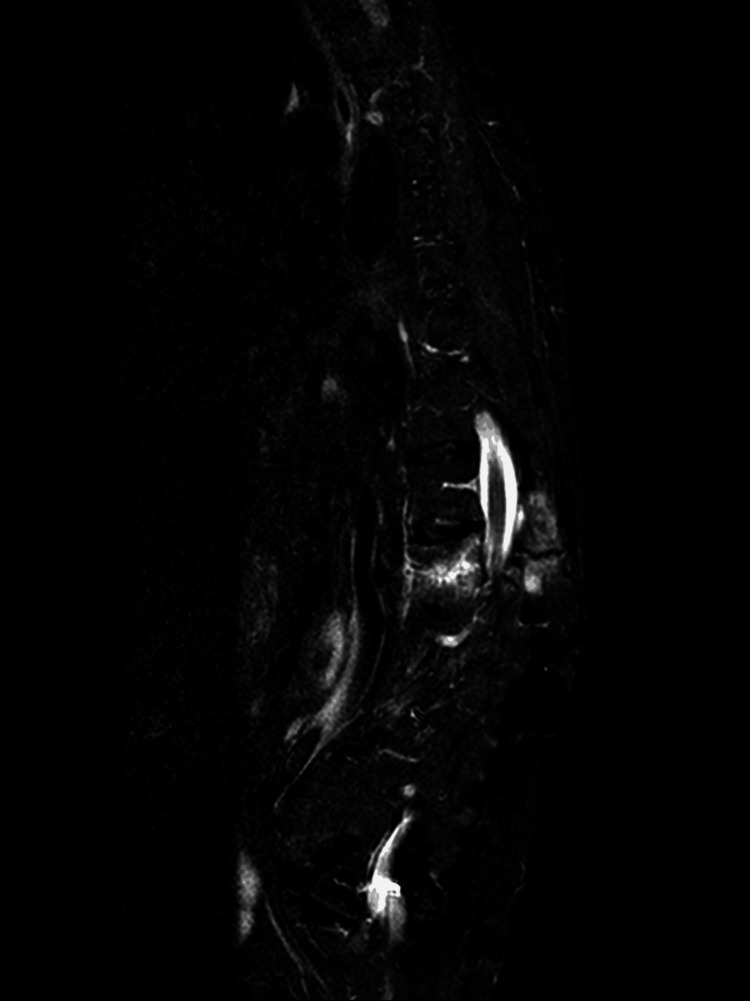
Sagittal STIR MRI sequence demonstrating hyperintense signal in the vertebral endplates of T12 and L1, consistent with acute bone marrow edema, as well as hyperintensity in the posterior column of the spine, indicating involvement of posterior elements. STIR: Short Tau Inversion Recovery; MRI: Magnetic Resonance Imaging

Sagittal non-contrast magnetic resonance imaging (MRI) using Short Tau Inversion Recovery (STIR) sequences (Figure 7) demonstrated altered signal intensity with hyperintensity in the vertebral endplates of T12 and L1, consistent with acute bone marrow edema, as well as in the posterior column of the spine, indicating active inflammation or injury.

A diagnosis of L1 vertebral body fracture, classified as AO 53.1.B2, was established, along with spinal deformity secondary to prior instrumentation for idiopathic scoliosis. This classification was supported by imaging findings, most notably the discontinuity of the articular facets at T12, L1, and L2, which indicates osseous disruption of the posterior column, consistent with a flexion-distraction injury mechanism. Additionally, the presence of posterior bone marrow edema on STIR sequences reinforces this interpretation. The collapse of the L1 vertebral body, with approximately 70% height loss, reflects a significant axial load component. Notably, the preexisting spinal deformity complicates the interpretation of the injury mechanism, as anatomical distortions and altered biomechanical dynamics due to prior instrumentation can obscure classical fracture patterns. This highlights the importance of advanced imaging and careful assessment to accurately characterize the lesion and optimize surgical decision-making.

**Figure 8:**
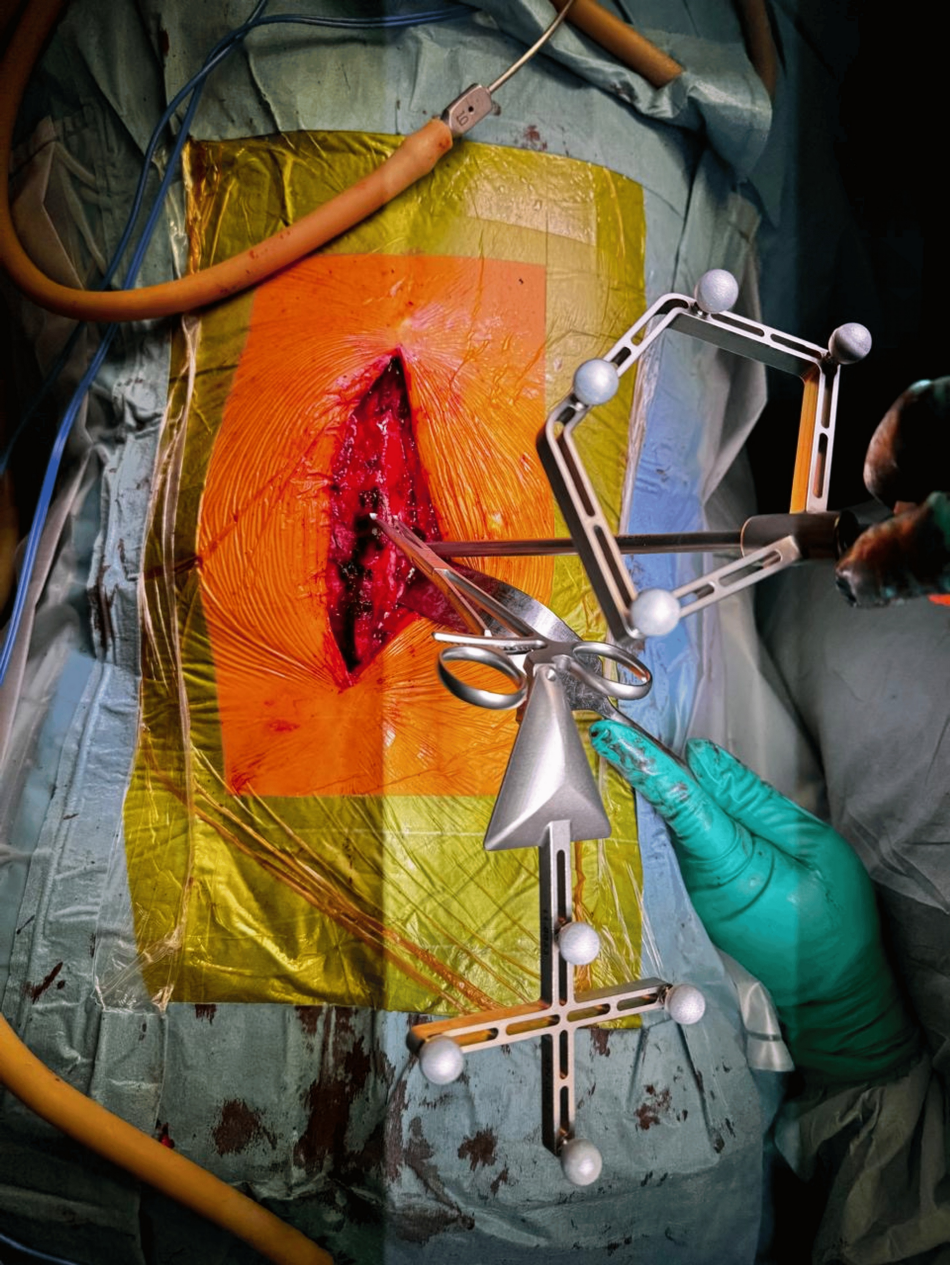
Intraoperative image showing the tracked pointer used for anatomical registration with the 7D Flash™ Navigation System (SeaSpine, Carlsbad, CA, USA).

**Figure 9:**
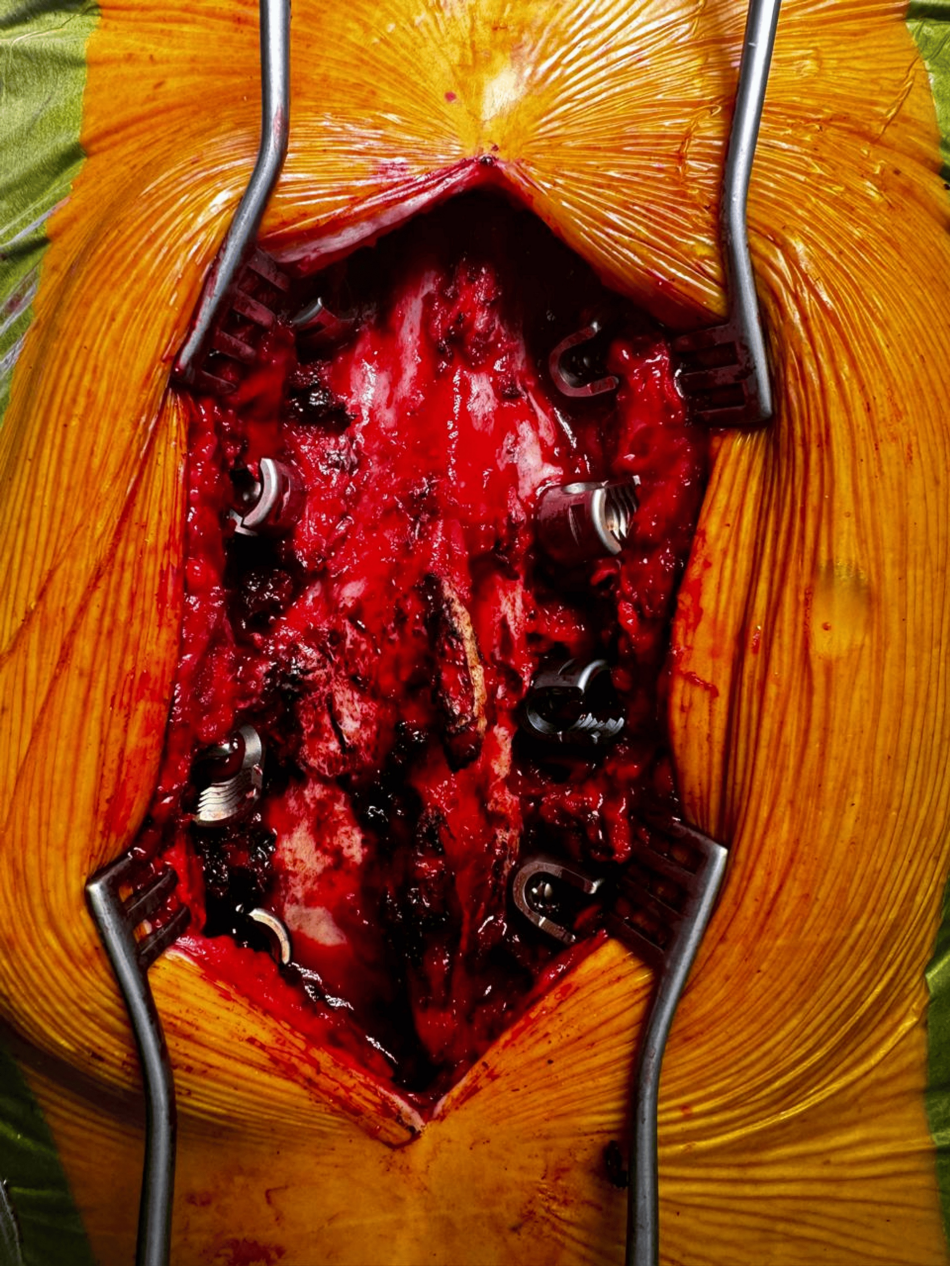
Intraoperative photograph demonstrating exposed pedicle screw heads from T12 to L3 prior to rod placement.

A posterior open approach was utilized for thoracolumbar transpedicular instrumentation, guided by the 7D Flash™ Navigation System (SeaSpine, Carlsbad, CA, USA). Anatomical registration was performed using a tracked pointer (Figure 8), allowing the system to localize the patient's anatomy without the need for intraoperative CT. Real-time navigation facilitated precise alignment and trajectory planning of pedicle screws based on the preoperative CT template. This technology enabled confirmation of accurate screw placement and provided valuable metadata for each screw, enhancing intraoperative decision-making and ensuring optimal implant positioning. Instrumentation was placed according to preoperative planning, and a subsequent intraoperative view captured the exposed screw heads across the construct before placement of the longitudinal connecting rods (Figure 9).

Instrumentation was placed from T12 to L3 and secured with longitudinal rods and a transverse connector. This construct aimed to restore sagittal alignment, ensure segmental stability, and minimize the risk of postoperative displacement or hardware-related complications.

**Figure 10:**
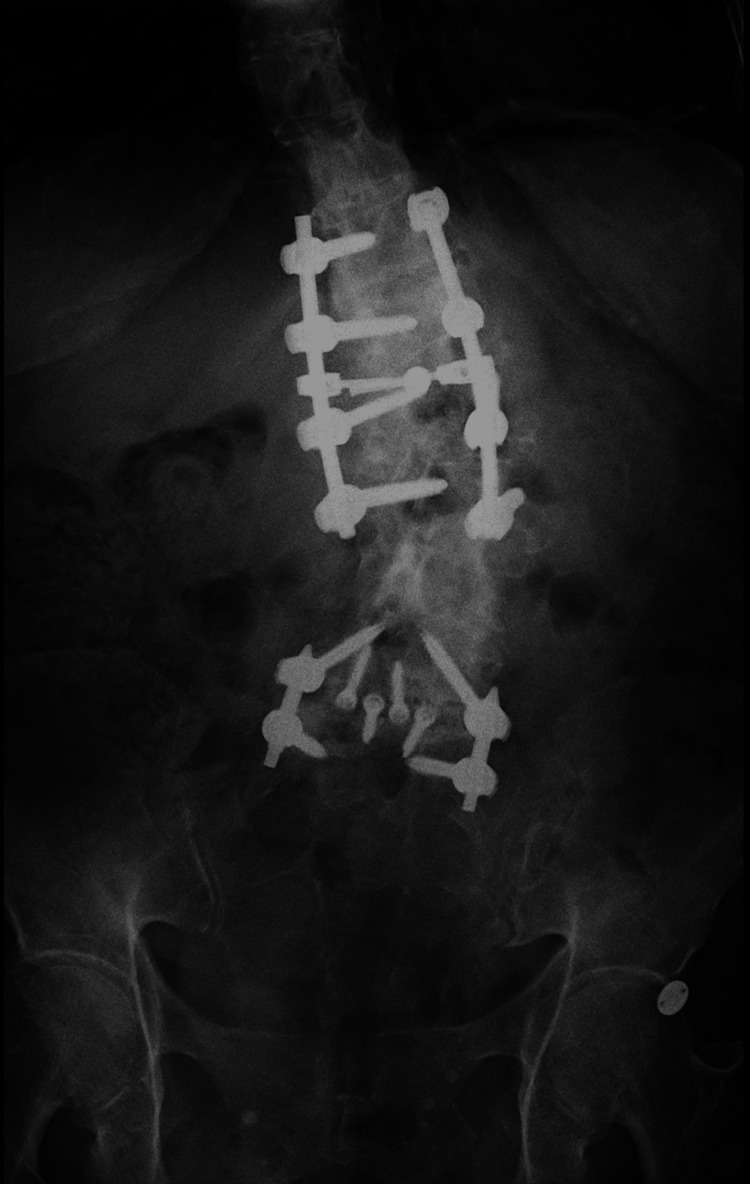
AP radiograph of the thoracolumbar spine demonstrating well-positioned pedicle screws from T12 to L3 without signs of loosening. AP: Anteroposterior

**Figure 11:**
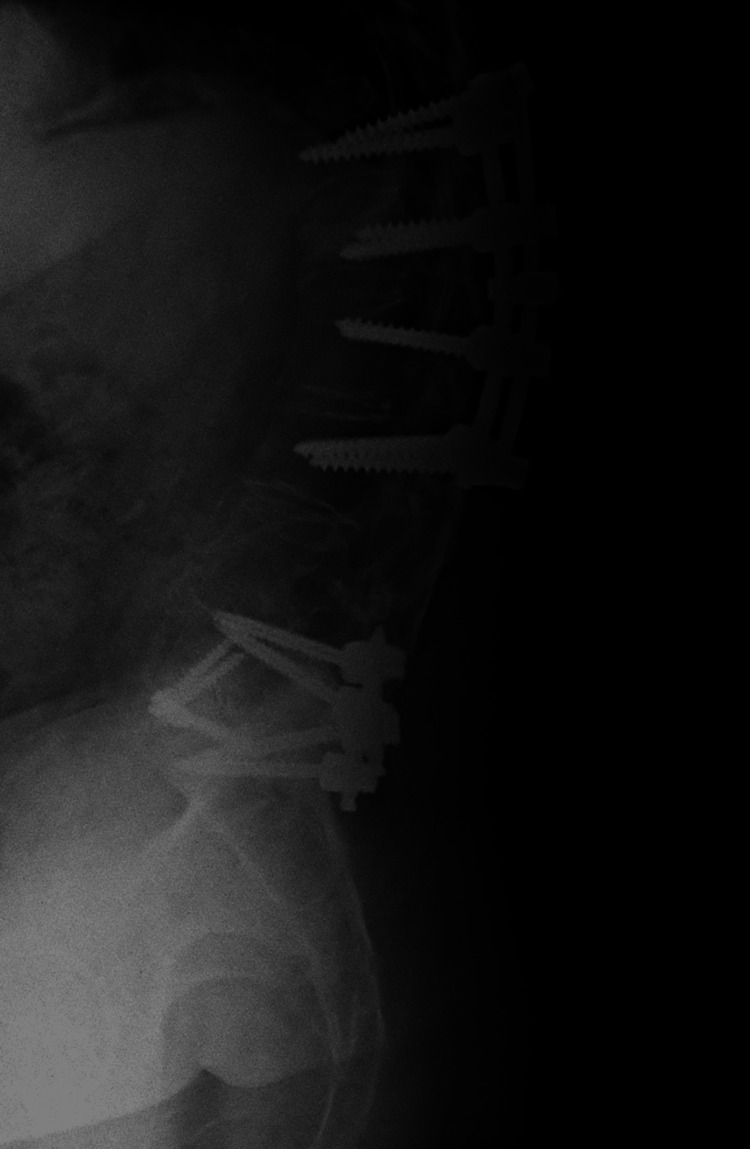
Lateral radiograph of the thoracolumbar spine showing proper pedicle screw alignment and preserved lumbar lordosis.

**Figure 12:**
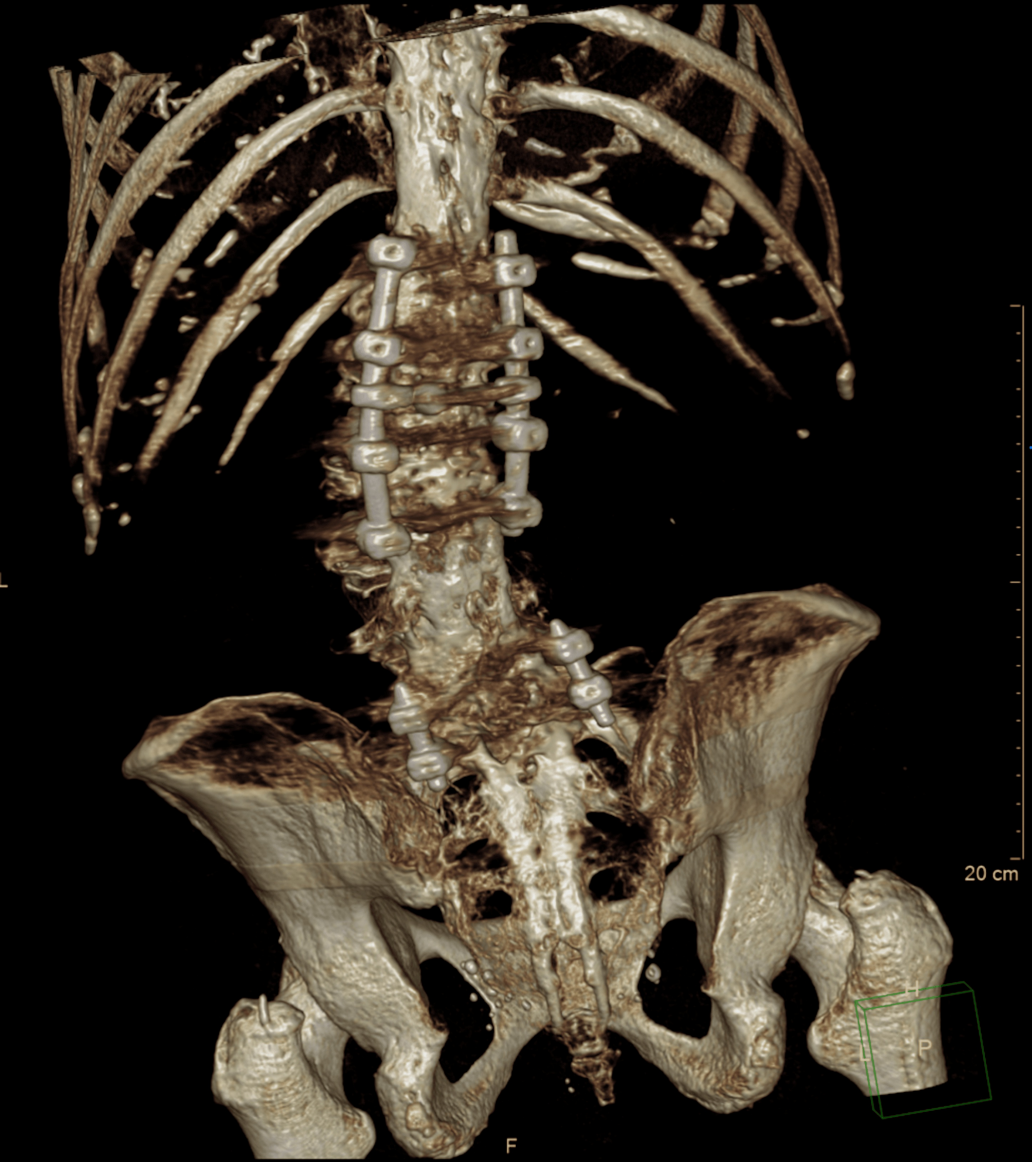
Three-dimensional CT reconstruction of the thoracolumbar spine, confirming accurate hardware placement without complications.

Postoperative imaging obtained during the first postoperative follow-up demonstrated satisfactory instrumentation and alignment. Figure 10 shows an anteroposterior radiograph of the thoracolumbar spine, revealing bilateral pedicle screws from T12 to L3 with symmetrical distribution, appropriate depth, and no radiographic signs of loosening. Figure 11 displays a lateral radiograph confirming the correct sagittal alignment and screw trajectory, with preservation of lumbar lordosis. Figure 12 presents a three-dimensional CT reconstruction showing intact instrumentation with proper placement and no evidence of mechanical complications.

Additionally, Table 1 provides a morphometric analysis of the relationship between pedicle screw characteristics and pedicle dimensions from T12 to L3. The SD/PW ratio assesses the proportionality between screw size and pedicle diameter, which is critical for safe and accurate screw placement. Table 2 analyzes the pedicle screw length within the vertebral body from T12 to L3, showing the vertebral body length, screw length inside the vertebral body, and the percentage of the vertebral body occupied by the pedicle screw, which is important for assessing optimal screw placement and fixation stability.

**Table 1:** Morphometric analysis of pedicle screw and pedicle dimensions from T12 to L3, including the SD/PW ratio as a key metric for screw fit. SD: Screw Diameter; SL: Screw Length; PW: Pedicle Width; PL: Pedicle Length; SD/PW: Screw Diameter/Pedicle Width Ratio

Level (R/L)	SD (mm)	SL (mm)	PW (mm)	PL (mm)	SD/PW
T12 R	6.5	45	9.36	44.98	0.69
T12 L	6.5	40	7.82	44.74	0.83
L1 R	6.5	45	8.01	52.25	0.81
L1 L	6.5	40	9.18	50.95	0.7
L2 R	6.5	45	6.82	58.84	0.95
L2 L	6.5	45	7.12	60.06	0.91
L3 R	6.5	50	6.82	56.82	0.95
L3 L	6.5	45	6.9	60.15	0.94

**Table 2:** Analysis of pedicle screw length relative to vertebral body dimensions from T12 to L3, including the percentage of vertebral body occupied by the screw. VB Length: Vertebral Body Length; SL in VB: Screw Length within Vertebral Body; % SL in VB: Percentage of Vertebral Body Length Occupied by the Pedicle Screw

Level (R/L)	VB Length (mm)	Screw Length in VB (mm)	% Screw in VB
T12 R	28.5	20.88	73.30%
T12 L	28.5	24.3	85.30%
L1 R	29.65	20.74	70.00%
L1 L	29.65	17.78	60.00%
L2 R	31.76	26.3	82.80%
L2 L	31.76	15.11	47.60%
L3 R	32.98	26.13	79.20%
L3 L	32.98	20.75	62.90%

## Discussion

This case illustrates the complex interplay between prior spinal instrumentation, mechanical failure, and the utility of navigation-assisted surgery in restoring spinal stability and alignment. The patient presented with a thoracolumbar fracture at L1 in the setting of long-standing postsurgical deformity secondary to AIS correction, compounded by multiple prior interventions, including decompression and ALIF. Such anatomical and biomechanical alterations are not uncommon in aging patients with a history of extensive spinal surgery, where segmental degeneration, junctional stress, and structural fatigue may converge to produce delayed instability or fractures, even in the absence of high-energy trauma [3].

The decision to perform posterior thoracolumbar transpedicular instrumentation from T12 to L3 was guided by the need to span the injured segment and anchor to structurally competent vertebrae while restoring sagittal alignment. Notably, the use of image-guided navigation was instrumental in achieving safe and precise screw placement, especially in a spine that had previously undergone multiple interventions. Navigation enabled real-time trajectory planning using preoperative CT images, obviating the need for additional intraoperative CT scans, reducing radiation exposure while enhancing surgical accuracy.

The 7D Flash™ Navigation System (SeaSpine) allowed for streamlined registration and accurate pedicle screw placement in segments with distorted anatomy. This is particularly relevant in revision surgery, where conventional anatomical landmarks are often obscured or altered by scar tissue, prior hardware, or deformity. As Tian et al. describe, CT-based navigation provides multiplanar visualization that is not hindered by abnormal structures, offering critical guidance in cases where fusion masses obscure tactile feedback and anatomical references [6]. Recent studies have further reported that intraoperative navigation not only increases screw placement accuracy but also facilitates the use of larger-diameter screws with improved biomechanical profiles (i.e., higher SD/PW ratios), thereby contributing to greater construct stability [2].

In addition to diameter, optimal screw length is also critical for maximizing fixation strength without breaching the anterior cortex. As Chua et al. demonstrated in a CT-based evaluation of 771 screws, ideal screw lengths range approximately from 88% of the vertebral body length at L1 to 79% at L5, with excessive depth increasing the risk of anterior cortical violation and insufficient depth reducing pullout strength [7]. While many studies support the superiority of navigation-assisted techniques, Kosmopoulos and Schizas highlighted that, in a meta-analysis of in vivo data, the weighted mean accuracy of pedicle screw placement was nearly identical between navigation-assisted (90.6%) and non-assisted (90.7%) subgroups [8]. They also noted a lack of standardized assessment methods across studies, cautioning against overgeneralization of accuracy metrics. This underscores the need to interpret such data carefully and within the broader clinical context.

Furthermore, this case underscores the clinical relevance of stress shielding and bone remodeling around long-standing instrumentation. The observed vertebral body collapse at L1, located at the junction between previous and recent hardware, aligns with reports suggesting that stress redistribution and bone demineralization at transitional zones may predispose to insufficiency fractures [3]. This pathomechanism necessitates a careful preoperative analysis of load-bearing dynamics and highlights the value of extending constructs beyond transition zones when necessary to prevent further failure.

The use of navigation in this context also supported decision-making intraoperatively, allowing for precise planning despite abnormal facet orientations, pedicle asymmetry, and prior bone remodeling. The real-time feedback and metadata provided by the navigation system ensured not only technical accuracy but also intraoperative confidence in achieving robust fixation.

Although navigation does not eliminate the complexity inherent to revision spine surgery, its integration can substantially mitigate intraoperative risk, reduce radiation exposure, and improve implant optimization. Nonetheless, as emphasized by Tjardes et al., long-term implementation of image-guided systems requires not only technical proficiency but also the preservation of skills in conventional (rescue) techniques to address intraoperative challenges when navigation is limited or unavailable [9]. In patients with a history of AIS correction and multiple subsequent procedures, the benefits of navigation are especially relevant, given the anatomical distortion and altered biomechanics that complicate surgical planning and execution.

## Conclusions

This case highlights the value of computer-assisted navigation in managing complex spinal pathology, particularly in patients with a history of AIS and prior instrumentation. The integration of the 7D Flash™ Navigation System (SeaSpine) enabled precise pedicle screw placement in a region of altered anatomy and mechanical compromise, minimizing the risk of neurovascular injury and optimizing construct stability. Given the presence of a postsurgical flat back deformity and an L1 vertebral body fracture with posterior column disruption, image-guided navigation proved critical in planning and executing safe trajectories within a limited surgical corridor. While further studies are needed to evaluate long-term outcomes, this case supports the expanding role of spinal navigation in revision and deformity surgery, especially in anatomically challenging scenarios where conventional techniques may fall short. Navigation should be considered an essential tool in the modern spine surgeon's armamentarium, contributing to both technical accuracy and patient safety.
